# Stereotactic Radiotherapy for Cervical Spinal Intramedullary Metastasis and Multiple Brain Metastases: A Case Report

**DOI:** 10.7759/cureus.590

**Published:** 2016-04-27

**Authors:** Yoshimasa Mori, Toshiki Kawamura, Yukihiko Ohshima, Arisa Takeuchi, Toshie Mori, Tuneo Ishiguchi

**Affiliations:** 1 Department of Radiology and Radiation Oncology, Aichi Medical University

**Keywords:** Stereotactic Radiosurgery, stereotactic radiotherapy, volumetric modulated arc therapy (vmat), spine metastasis, brain metastasis, dynamic conformal arc therapy, intramedullary spinal cord metastases, true beam s tx, papillary thyroid carcinoma, simultaneous integrated boost

## Abstract

A case of cervical (C) spinal intramedullary metastasis and multiple small brain metastases from papillary thyroid carcinoma was presented. Spinal metastasis caused posterior neck and left shoulder pain, dysesthesia in both legs, and motor weakness in both legs and left arm, though the brain metastases were asymptomatic. Both the spinal and brain metastases were successfully treated by frameless stereotactic radiotherapy (SRT)/stereotactic radiosurgery (SRS). The patient's symptoms were almost entirely relieved within two months.

A 76-year-old woman was diagnosed as having a thyroid tumor and lung metastasis by roentgenography and computed tomography. Biopsy of the thyroid tumor extending into the mediastinum revealed papillary thyroid carcinoma. She underwent surgical resection of thyroid with dissection of the mediastinum lymph node area. Internal oral radioisotope therapy was not effective for the multiple small lung metastases. She did well for 15 months, but later developed posterior neck and left shoulder pain and dysesthesia in the right leg and then dysesthesia and motor weakness in both legs. Then she experienced weakness in the left upper extremity. Magnetic resonance imaging (MRI) disclosed a small cervical spinal intramedullary mass lesion at the level of C6 and C7 on the left side as well as nine small brain lesions. The cervical spinal intramedullary metastatic tumor was treated by volumetric modulated arc radiotherapy (VMAT) SRT and the nine small brain metastatic tumors were treated by dynamic conformal arc (DCA) SRS uneventfully. A total dose of 39 Gy (100% dose) was delivered in 13 fractions for the spinal lesion (prescription, D95=95% dose; maximum dose=46.3 Gy). Single fraction SRS of 22 Gy (prescription, D95=100% dose) was performed for each of the nine small brain tumors. The spinal tumor was decreased in size on follow-up MRI two months after SRT. Three of the nine brain lesions had disappeared and six were decreased in size on follow-up MRI two months after SRS. Motor weakness in the left extremities and right leg was fully improved, and she could walk again without a cane within two months after SRT. She had only slight dysesthesia in the right leg, possibly due to lumbar spondylosis at the end of the six-month follow-up after SRT. The spinal tumor continued to decrease in size on follow-up MRI five months after SRT. Eight of the nine brain lesions had disappeared and one was decreased in size on follow-up MRI five months after SRS.

## Introduction

A case of cervical (C) spinal intramedullary metastasis and multiple small brain metastases from papillary thyroid carcinoma was presented. Spinal metastasis caused posterior neck and left shoulder pain, dysesthesia in both legs, and motor weakness in both legs and left arm, though the brain metastases were asymptomatic. The single spinal lesion and nine brain lesions were successfully treated by frameless stereotactic radiotherapy (SRT) and stereotactic radiosurgery (SRS). The patient's symptoms were almost completely relieved after treatment.

Many reports have been published describing the effectiveness of SRT/SRS for small brain parenchymal metastases [1]. However, few reports are available on SRT/SRS for spinal intramedullary metastases [2-4]. The low tolerance of the spinal cord to radiation often limits the treatment dose in conventional external beam radiotherapy (EBRT) to a level below the optimal tumor treatment dose, because radiation myelopathy can result in severe functional deficits. The newer technique of SRT/SRS accurately concentrates a high dose on the tumor while simultaneously sparing surrounding structures. In this case, SRT with volume modulated arc radiotherapy (VMAT) was performed for the cervical spinal intramedullary lesion and SRS with dynamic conformal arc (DCA) was done for the brain lesions successfully. Informed consent was obtained from the patient for this study.

## Case presentation

**Figure 1:**
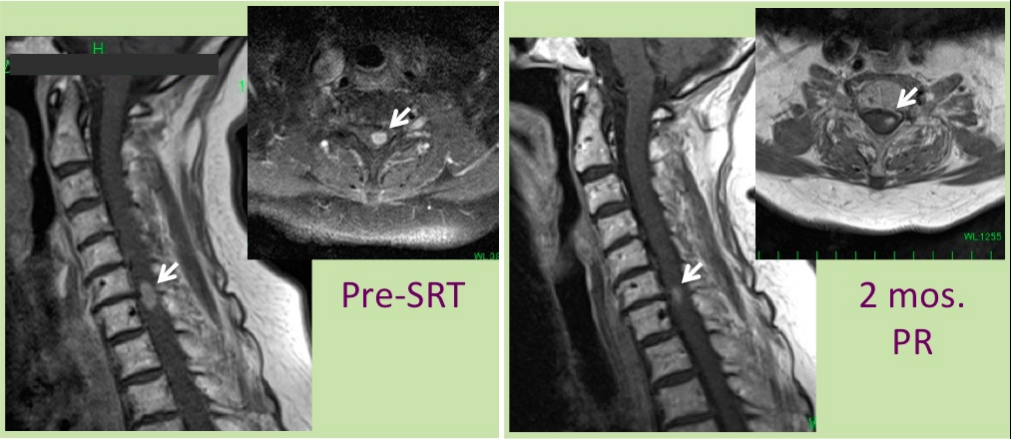
Pre-SRT and post-SRT MRIs of cervical spinal lesion *Left *: sagittal and axial view of gadolinium (Gd) enhanced magnetic resonance images (MRI) before stereotactic radiotherapy (SRT). *Right*: sagittal and axial view two months after SRT. A spinal intramedullary lesion (arrows) at the level of cervical (C) 6th and 7th vertebra shrank within two months after volumetric arc modulated therapy (VMAT) SRT.

A 76-year-old woman was diagnosed as having a thyroid tumor and lung metastasis by roentgenography and computed tomography at a health check. Biopsy of the thyroid tumor extending into the mediastinum revealed papillary thyroid carcinoma. She underwent surgical resection of the thyroid with dissection of the mediastinum lymph node area. Internal oral radioisotope therapy was not effective for the multiple small lung metastases. She did well for 15 months, but later she developed posterior neck and left shoulder pain and dysesthesia in the right leg and then dysesthesia and motor weakness in both legs. Then, she experienced weakness in the left upper extremity. Magnetic resonance imaging (MRI) disclosed a small cervical spinal intramedullary mass lesion at the level of C6 and C7 on the left side (Figure 1).

**Figure 2:**
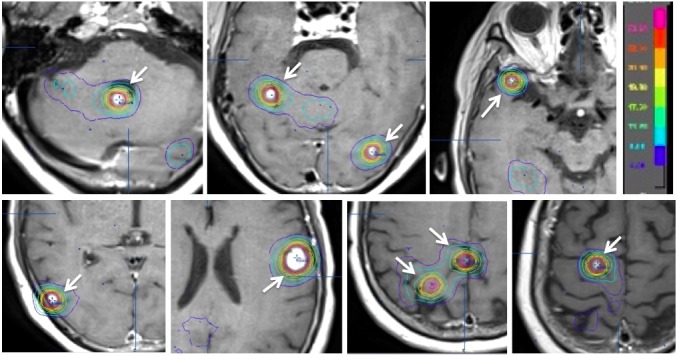
Pre-SRS MRI of brain lesions (Dose planning on iPlan) Axial MRI with Gd enhancement on iPlan (BrainLAB, Tokyo) radiation therapy planning system (RTPS) workstation. Nine small brain lesions (arrows) were treated by single session stereotactic radiosurgery (SRS). Each lesion was targeted with four arcs by dynamic conformal arc (DCA). 100% dose=22 Gy (single fraction SRS), D95=100%dose.

In addition, nine small brain lesions were observed (Figure 2).

**Figure 3:**
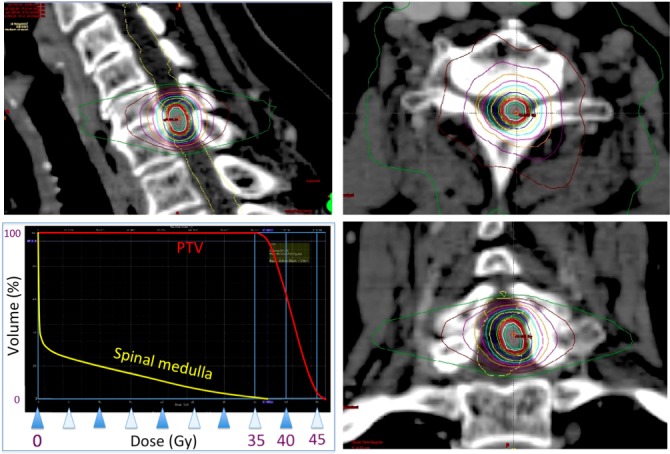
Pre-SRT CT of cervical lesion (Dose planning on Eclipse) Sagittal (*left upper*), axial (*right upper*), and coronal (*right lower*) images of iodine enhancement computed tomography (CT) on Eclipse (Varian, Tokyo) RTPS workstation. Dose-volume histogram is also shown (*left lower*). The enhanced intramedullary lesion was targeted with excellent conformity by VMAT simultaneously with sparing of the surrounding normal spinal medulla (*See also pre-SRT axial MRI of Figure 1 left*). A simultaneous boost up to 46.3 Gy was performed inside the tumor. 100% dose=39 Gy in 13 fractions, D95=95% dose.

The cervical spinal intramedullary metastatic tumor was treated by VMAT SRT and the nine small brain metastatic tumors were treated by DCA SRS, using TrueBeam STx (Varian, Tokyo) uneventfully. A total dose of 39 Gy (100% dose) was delivered in 13 fractions with two coplanar full rotation method around one isocenter for the spinal lesion (prescription, D95 [dose to 95% volume of target]= 95% dose; maximum dose=46.3 Gy) (Figure 3).

Later, single fraction SRS of 22 Gy (prescription, D95=100% dose=22 Gy) was performed for each of the nine small brain tumors in three-day therapy, in which three tumors each were treated per day. The spinal tumor had decreased in size on follow-up MRI two months after SRT. Three of the nine brain lesions had disappeared and six were decreased in size on follow-up MRI two months after SRS. Motor weakness in the left extremities and right leg was fully improved, and she could walk again without a cane within two months after SRT. She had only slight dysesthesia in the right leg, possibly due to lumbar spondylosis at the end of the six-month follow-up after SRT. The decrease in size of the spinal tumor was sustained on follow-up MRI five months after SRT. Eight of the nine brain lesions had disappeared and one was decreased in size on follow-up MRI five months after SRS. Thoracic, lumbar, and sacral spine were intact on MRI inspection throughout the course.

## Discussion

SRT/SRS needs an accurate technique to safely concentrate radiation on the target. TrueBeam equipped with ExacTrac system uses X-ray image analysis to correct patient position before each treatment session. A spinal lesion is accurately targeted after localization of the spinal bone structures. The spinal cord is spared as much as possible, while the tumor receives a higher dose than possible with conventional EBRT. Some reports have noted successful results of SRT/SRS for spinal metastases, but most describe only spinal bone metastases. Few reports have focused on the results of SRT/SRS for spinal intramedullary metastases [2-4].

Endo et al. [5] reviewed reports of conventional EBRT for intramedullary spinal cord metastases, and found that a total dose of 25 to 40 Gy improved patients' symptoms in 84.2% (116 of 191). Shin et al. [2] reported treatment results of spinal SRS for intramedullary metastases in six patients (six tumors). The treatment dose was 10–16 Gy. They observed that all tumors except for one without imaging follow-up were controlled without any adverse effects noted. Parikh et al. [3] reported a case of C5 intramedullary spinal cord metastasis. The tumor was resistant to conventional EBRT of 30 Gy in 10 fractions. As a retreatment, CyberKnife SRT with a total dose of 15 Gy in three fractions (margin dose at 80% isodose line) successfully shrank the tumor and improved the patient’s symptoms until the end of the follow-up period 26 months after SRT. Mori et al. [4] reported a case of cervical spinal intramedullary metastatic lesions in C1 and C2. The C1 lesion was inside the field of the previous whole brain radiotherapy of 40 Gy in 20 fractions for multiple brain metastases and C2 lesion was just outside the field. A total dose of 24 Gy (at 100% isodose) in eight fractions was delivered for C1 lesion and 36 Gy in 12 fractions (at 100% isodose) was delivered to the C2 lesion using a multi-circular cone collimator method. They described that both tumors were controlled until the patient’s death from primary lung carcinoma 10 months after SRT. The patient’s neurological symptom of mild ataxia was stable until his death.

The tolerance dose (TD) to the spinal cord is usually quoted as 45 to 50 Gy in 2-Gy fractions, which is known to be TD 5/5, with 5% severe complication probability in five years [6]. However, more recent studies that included large numbers of patients have shown that a more realistic TD 5/5 could be up to 60 Gy [7]. Sahgal et al. [8] found that a dose of approximately 70 Gy or less, in a total maximum point dose normalized to a 2-Gy equivalent dose, was safe. Recently Park et al. [9] reviewed SRT/SRS for intramedullary spinal lesions. They summarized relatively low doses for the safe dose to a point within the thecal sac. They also mentioned that the decision to use higher doses must weigh the benefit of tumor control against the potential for radiation toxicity.

In the present case, VMAT SRT was performed for a spinal lesion entirely surrounded by normal spinal medulla. Arc radiation delivery of VMAT is thought to be better than static multi-beam, because an increased dose area beside the target is less likely. A total dose of 39 Gy in 13 fractions is almost equivalent to 50 Gy in 25 fractions for spinal tolerance. A 3-Gy fraction schedule was adopted because fractionation with a reasonable treatment period would help tolerance of the surrounding spinal medulla without exceeding the dose to the medulla just beside the tumor caused by tumor shrinkage in the case of a longer treatment period. Around the tumor border and surrounding spinal medulla a total dose of 95% of 39 Gy in 13 fractions was delivered. Simultaneously a greater boost dose up to 46.3 Gy in 13 fractions was given to the interior of the tumor. Boost dose inside the tumor might contribute to quick shrinkage of the tumor. This strategy with ‘reasonable margin dose and more central dose’ by VMAT is compatible to CyberKnife SRT such as that used by Parikh et al. [3]. Though the follow-up period is not long, the tumor decreased in size successfully and the patient’s symptoms were improved relatively quickly in the present case. This patient had lung metastases as well, but papillary thyroid carcinoma is usually slow-growing. Long-term follow-up is necessary.

## Conclusions

Though the follow-up period is not long, VMAT SRT was effective in controlling a cervical spinal intramedullary metastasis and improving the patient’s symptoms. DCA SRS was also effective in controlling her small brain metastases.
